# Monocular Stereo Measurement Using High-Speed Catadioptric Tracking

**DOI:** 10.3390/s17081839

**Published:** 2017-08-09

**Authors:** Shaopeng Hu, Yuji Matsumoto, Takeshi Takaki, Idaku Ishii

**Affiliations:** Department of System Cybernetics, Hiroshima University, 1-4-1 Kagamiyama, Higashi-Hiroshima, Hiroshima 739-8527, Japan; hu@robotics.hiroshima-u.ac.jp (S.H.); yuji-matsumoto@hiroshima-u.ac.jp (Y.M.); takaki@robotics.hiroshima-u.ac.jp (T.T.)

**Keywords:** high-speed vision, stereo tracking, catadioptric stereo, viewpoint switching, multithread active vision

## Abstract

This paper presents a novel concept of real-time catadioptric stereo tracking using a single ultrafast mirror-drive pan-tilt active vision system that can simultaneously switch between hundreds of different views in a second. By accelerating video-shooting, computation, and actuation at the millisecond-granularity level for time-division multithreaded processing in ultrafast gaze control, the active vision system can function virtually as two or more tracking cameras with different views. It enables a single active vision system to act as virtual left and right pan-tilt cameras that can simultaneously shoot a pair of stereo images for the same object to be observed at arbitrary viewpoints by switching the direction of the mirrors of the active vision system frame by frame. We developed a monocular galvano-mirror-based stereo tracking system that can switch between 500 different views in a second, and it functions as a catadioptric active stereo with left and right pan-tilt tracking cameras that can virtually capture 8-bit color 512×512 images each operating at 250 fps to mechanically track a fast-moving object with a sufficient parallax for accurate 3D measurement. Several tracking experiments for moving objects in 3D space are described to demonstrate the performance of our monocular stereo tracking system.

## 1. Introduction

Stereo vision is a range-sensing technique for distant real-world scenes using multiple images observed at different viewpoints with triangulation, and many stereo matching algorithms have been reported for stereo disparity map estimation [1,2,3,4,5,6,7,8]; they are classified into (1) global algorithms to perform global optimization for the whole image to estimate the disparity of every pixel with numerical methods [9,10,11,12,13,14], and (2) local algorithms with window-based matching that only requires local image features in a finite-size window when computing disparity at a given point with the winner-take-all strategy [15,16,17,18,19,20,21]. Compared with accurate but time-consuming global algorithms, local algorithms are much less time-consuming in estimating disparity maps, and therefore many real-time stereo systems capable of executing local algorithms have been reported, such as Graphic Processing Unit (GPU)-based stereo matching [22,23,24,25,26] and Field Programmable Gate Array (FPGA)-based embedded systems [27,28,29,30].

For a wider field of view without decreasing resolution, many active stereo systems that mount cameras on pan-tilt mechanisms have been reported [31,32,33,34]; they are classified into (1) multiple cameras on a single pan-tilt mechanism; and (2) multiple pan-tilt cameras, on which each camera has its pan-tilt mechanism. In the former approach, the relative geometrical relationship between cameras are fixed in a way that the camera parameters can be easily calibrated for stereo measurement; its measurable range in depth is limited because the vergence angle between cameras is fixed. The latter approach can expand the measurable range in the depth direction, as well as those in the pan and tilt directions, because the vergence angle between cameras can be freely controlled; the camera parameters should be calibrated for accurate stereo measurement frame by frame according to the time-varying vergence angle in stereo tracking. With the recent spread of distributed camera networks for wide-area video surveillance, many studies that concern gaze control [35,36,37], camera calibration [38,39,40,41,42,43], and image rectification in stereo matching [44,45], for the latter approach, have been reported for stereo tracking using multiple PTZ (pan-tilt-zoom) cameras located at different sites. The pros and cons of the stereo vision techniques and the active stereo systems are summarized in Table 1.

To reduce the complexity in calibration of camera parameters when using multiple cameras, many monocular stereo methods have been proposed; they are classified into (1) motion stereo that calculates range information from multiple images captured at different timings [47,48,49]; (2) image layering stereo that incorporates multi-view information in a single image via a single-lens aperture [50] and coded aperture [51,52]; and (3) catadioptric stereo for which a single image involves mirror-reflected multi-view data; the camera’s field of view is divided either by a single planar mirror [53,54], two or three planar mirrors [55,56,57], four planar mirrors [58,59], bi-prism mirrors [60,61], or convex mirrors [62,63,64,65,66,67]. Motion stereo can freely set the baseline width and vergence angle between virtual cameras at different timings for accurate stereo measurement, whereas it is limited to measuring stationary scenes due to the synchronization error caused by the delay time among multiple images. The image-layering stereo requires a decoding process to extract multi-view data from a single image; it is limited in accuracy due to the very narrow baseline width of stereo measurement on their designed apertures. Corresponding to the number of viewpoints, the catadioptric stereo has to divide the camera’s field of view into smaller fields for multi-view, whereas it can provide a relatively long baseline width and large vergence angle between mirrored virtual cameras for accurate stereo measurement. Considering camera calibration and stereo rectification [68,69], several real-time catadioptric stereo systems have also been developed [70,71,72,73,74,75]. However, most catadioptric stereo systems have not been used for an active stereo to expand the field of view for wide-area surveillance. This is because catadioptric stereo systems involving large mirrors are too heavy to quickly change their orientations, and it is difficult to control the pan and tilt angles of mirrored virtual cameras independently. Monocular stereo systems that can quickly switch their viewpoints with dynamic changing apertures, such as a programmable iris with a liquid crystal device [76] and multiple pinhole apertures with a rotating slit [77], also have been reported as expansions of an image-layering stereo with designed apertures, whereas they have not considered an active stereo for a wide field of view due to a very narrow baseline stereo measurement.

Thus, in this study, we implement a monocular stereo tracking system that expands on a concept of catadioptric stereo with a relatively long-width baseline to an active stereo that can control the pan and tilt directions of mirrored virtual cameras for the wider field of view, and develop a mirror-based ultrafast active vision system with a catadioptric mirror system that enables a frame-by-frame viewpoint switching of pan and tilt controls of mirrored virtual tracking cameras at hundreds of frames per second. The remainder of this paper is organized as follows. Section 2 proposes a concept of catadioptric stereo tracking using multithread gaze control to perform as multiple virtual pan-tilt cameras, and the detail of its geometry is described in Section 3. Section 4 gives an outline of the configuration of our catadioptric stereo tracking system that can alternatively switch 500 different views in a second, and describes its implemented algorithms for catadioptric stereo tracking with virtual left and right pan-tilt cameras of 512×512 color images each operating at 250 fps. In Section 5, the effectiveness of our tracking system is verified by showing several range measurement results for moving scenes.

## 2. Catadioptric Stereo Tracking Using Multithread Gaze Control

**Figure 1:**
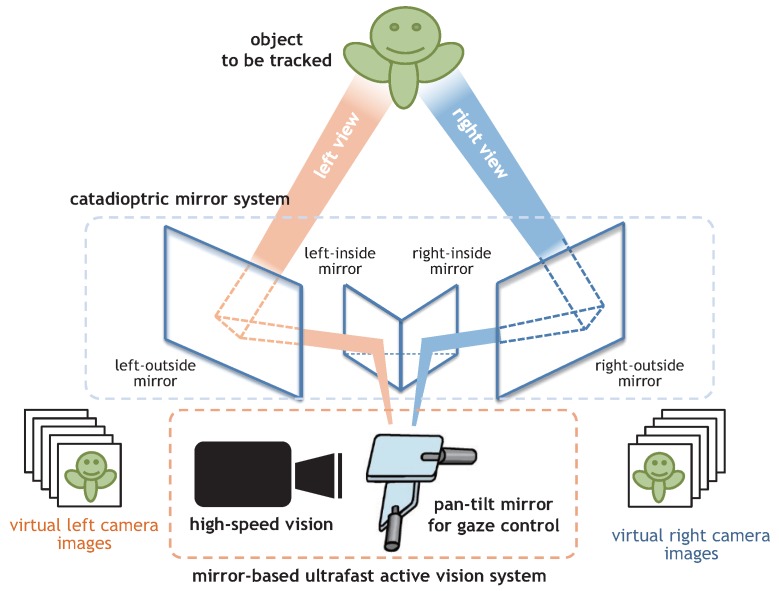
Catadioptric stereo tracking with multithread gaze control.

This section describes our concept of catadioptric stereo tracking on an ultrafast mirror-drive active vision system that can perform as two virtual pan-tilt cameras for left and right-side views by frame-by-frame switching the direction of the mirrors on the active vision system. Figure 1 shows the concept of catadioptric stereo tracking. Our catadioptric stereo tracking system consists of a mirror-based ultrafast active vision system and a catadioptric mirror system. The former consists of a high-speed vision system that can capture and process images in real time at a high frame rate, and a pan-tilt mirror system for ultrafast gaze control. It can be unified as an integrated pan-tilt camera and its complexity in system management is similar to those of standard PTZ cameras, which are commonly used in video surveillance applications. Figure 1 shows a catadioptric mirror system consisting of multiple planar mirrors on the left and right sides, and a pan-tilt mirror system installed in front of the lens of the high-speed vision system to switch between left- and right-side views by alternating the direction of its mirrors. The images on the side of the left-side mirror and the left half of the angle mirror are captured as the left-view images, and those on the side of the right-side mirror and the right half of the angle mirror are captured as the right-view images.

**Figure 2:**
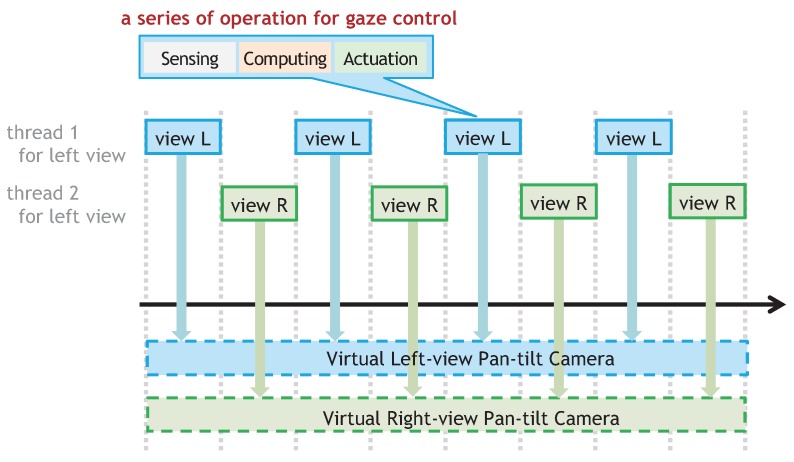
Time-division thread processes for virtual pan-tilt cameras in multithread gaze control.

Originating from multithreaded processing in which threads conducting tasks are simultaneously running on a computer using the time-sharing approach, our catadioptric stereo tracking method extends the concept of multithread gaze control to the pan-tilt camera by parallelizing a series of operation with video-shooting, processing, and gaze control into time-division thread processes with a fine temporal granularity to realize multiple virtual pan-tilt cameras on a single active vision system as illustrated in Figure 2. The following conditions are required so that a single active vision system with multithread gaze control has a potency equivalent to that of left and right pan-tilt cameras for accurate and high-speed stereo tracking with sufficient large parallax.

(1) Acceleration of video-shooting and processing

When left and right virtual pan-tilt cameras are shooting at the rate of dozens or hundreds of frames per second for tracking fast-moving objects in 3D scenes, the frame capturing and processing rate of an actual single vision system must be accelerated at a rate of several hundreds or thousands of frames per second to perform the video-shooting, processing, and tracking for left and right-view images of the virtual pan-tilt cameras.

(2) Acceleration of gaze control

To control the gaze of every frame independently, high-speed gaze control must ensure that a given frame does not affect the next frame. Corresponding to the acceleration of video-shooting and processing at a rate of several hundreds of frames per second, the temporal granularity of the time-division thread gaze control processes must be minimized at the millisecond level, and a high-speed actuator that has a frequency characteristic of a few kHz is required for acceleration of gaze control.

Compared with catadioptric systems with a fixed camera, catadioptric stereo tracking has the advantage of being able to mechanically track a target object as active stereo while zooming in the fields of views of virtual left and right pan-tilt cameras; multithread gaze control enables zoom-in tracking when the target is moving in the depth direction by controlling the vergence angle between two virtual pan-tilt cameras as well as when the target moves in the left-right or up-down direction. In catadioptric stereo tracking, correspondences among left and right-view images can be easily established because their camera internal parameters, such as focal length, gain, and exposure time, are the same in virtual left and right pan-tilt cameras, whose lens and image sensors are perfectly matched due to differences in their cameras’ internal parameters.

**Figure 3:**
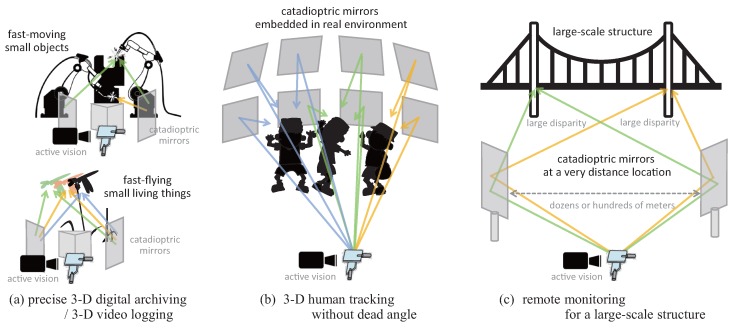
Configuration examples of catadioptric mirror systems, referring to practical applications of catadioptric stereo tracking: (**a**) precise 3-D digital archiving or 3-D video logging; (**b**) 3-D human tracking without dead angle; (**c**) remote monitoring for a large-scale structure.

The catadioptric mirror system in catadioptric stereo tracking can be designed flexibly in accordance with the requirements of its practical applications. Moreover, catadioptric stereo tracking has the following advantages over active stereo systems with multiple PTZ cameras: (1) space-saving installation that enables stereo measurement in a small space, where multiple PTZ cameras cannot be installed; (2) easy expandability for multi-view stereo measurement with a large number of mirrors; and (3) stereo measurement with arbitrary disparity without any electrical connection that enables precise long-distance 3D sensing. Figure 3 illustrates the configuration examples of the catadioptric mirror systems, referring to the practical applications of catadioptric stereo tracking: (a) precise 3D digital archiving/video logging for fast-moving small objects and creatures; (b) 3D human tracking without a dead angle, which functions as a large number of virtual pan-tilt cameras by utilizing multiple mirrors embedded in the real environment; and (c) remote surveillance for a large-scale structure with left and right mirrors at a distant location that requires a large disparity of dozens or hundreds of meters for precise 3D sensing. In this study, the catadioptric mirror system used in the experiments detailed in Section 5 was set up for a short-distance measurement to verify the performance of our catadioptric stereo tracking system in a desktop environment, corresponding to precise 3D digital archiving for fast-moving small objects.

Catadioptric stereo tracking has disadvantages: (1) inefficient use of incident light, owing to the small-size pan-tilt mirror, which is designed for ultrafast switching of viewpoints; and (2) synchronization errors in stereo measurement of moving targets, due to the delay time between virtual left and right-view images captured at different timings. These synchronization errors in catadioptric stereo tracking can be reduced by accelerating alternative switching of left and right views with multithreaded gaze control with a temporal granularity at the millisecond level. The pros and cons of the catadioptric systems with fixed mirrors, fixed camera systems, and our catadioptric stereo tracking system are summarized in Table 2.

## 3. Geometry of Catadioptric Stereo Tracking

This section describes the geometry of a catadioptric stereo tracking system that uses a pan-tilt mirror system with a single perspective camera and a catadioptric mirror system with four planar mirrors as illustrated in Figure 1, and derives the locations and orientations of virtual left and right pan-tilt cameras for triangulation in active stereo for time-varying 3D scenes.

### 3.1. Geometrical Definitions

#### 3.1.1. Pan-Tilt Mirror System

**Figure 4:**
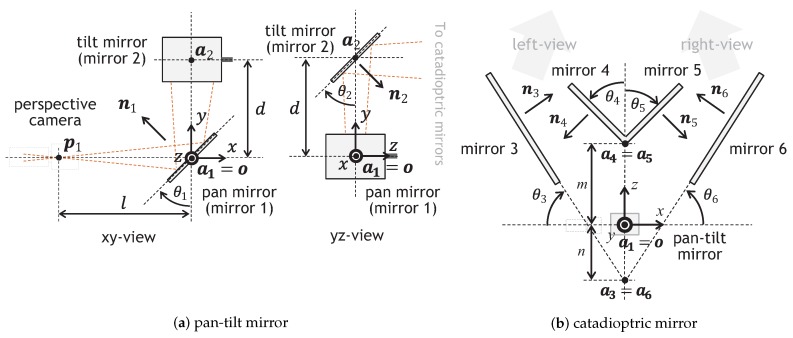
Geometries of the pan-tilt mirror system and the catadioptric mirror system: (**a**) pan-tilt mirror; (**b**) catadioptric mirror.

The pan-tilt mirror system assumed in this study has two movable mirrors in the pan and tilt directions: pan mirror and tilt mirror. Figure 4a shows the xy-view and yz-view of the geometrical configuration of the pan-tilt mirror system with a perspective camera; the xyz-coordinate system is set so that the *x*-axis corresponds to the optical axis of the camera, the *y*-axis corresponds to the line between the center points of the pan mirror and tilt mirror. The depth direction in stereo measurement corresponds to the *z*-direction. The center of the pan mirror (mirror 1) is set to $a_{1}={\left(0,0,0\right)}^{T}$, which is the origin of the xyz-coordinate system. The pan mirror can rotate around the *z*-axis, and its normal vector is given as $n_{1}={\left(-\cos\theta_{1},\sin\theta_{1},0\right)}^{T}$. The center of the tilt mirror (mirror 2) is located at $a_{2}={\left(0,d,0\right)}^{T}$, where its distance from that of the pan mirror is represented by *d*. The tilt mirror can rotate around a straight line parallel to the *x*-axis at a distance *d*, and its normal vector is given as $n_{2}={\left(0,-\sin\theta_{2},\cos\theta_{2}\right)}^{T}$. $\theta_{1}$ and $\theta_{2}$ indicate the pan and tilt angles of the pan-tilt mirror system, respectively. The optical center of the perspective camera is set to $p_{1}={\left(-l,0,0\right)}^{T}$, where its distance from the center of the pan mirror is represented by *l*.

#### 3.1.2. Catadioptric Mirror System

The catadioptric mirror system with four planar mirrors is installed in front of the pan-tilt mirror so that all the planar mirrors are parallel to the *y*-axis. Figure 4b shows the xz-view of its geometry. The locations of mirrors 3 and 4 for the left-side view and mirrors 5 and 6 for the right-side view are given. The normal vectors of the mirror planes i(=3,4,5,6) are given as $n_{3}={\left(\sin\theta_{3},0,\cos\theta_{3}\right)}^{T}$, $n_{4}={\left(-\cos\theta_{4},0,-\sin\theta_{4}\right)}^{T}$, $n_{5}={\left(\cos\theta_{5},0,-\sin\theta_{5}\right)}^{T}$, and $n_{6}={\left(-\sin\theta_{6},0,\cos\theta_{6}\right)}^{T}$, respectively. As illustrated in Figure 4b, $\theta_{3}$ and $\theta_{6}$ are the angles formed by the xy-plane and the planes of mirrors 3 and 6, respectively; $\theta_{4}$ and $\theta_{5}$ are those formed by the yz-plane and the planes of mirrors 4 and 5, respectively. A pair of mirrors 3 and 6 at the outside are located symmetrically with the yz-plane as well as a pair of mirrors 4 and 5 at the inside; $\theta_{3}=\theta_{6}$ and $\theta_{4}=\theta_{5}$. The planes of mirrors 4 and 5, and those of mirrors 3 and 6 are crossed on the yz-plane, respectively; their crossed lines pass through the points $a_{4}=\left(0,d,m\right)\left(=a_{5}\right)$ and $a_{3}=\left(0,d,n\right)\left(=a_{6}\right)$ in front of or behind the center of the tilt mirror, respectively.

### 3.2. Camera Parameters of Virtual Pan-Tilt Cameras

#### 3.2.1. Mirror Reflection

**Figure 5:**
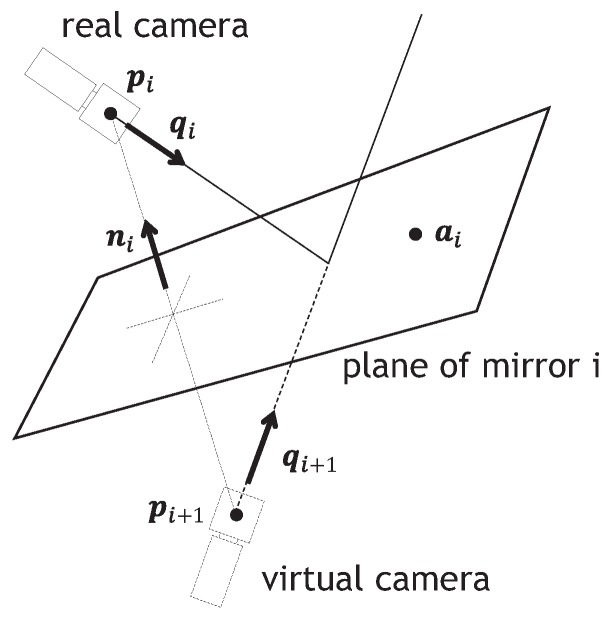
Relationship between real camera and its virtual camera reflected by a planar mirror.

The camera parameters of a virtual pan-tilt camera, whose optical path is reflected on a pan-tilt mirror system and a catadioptric mirror system multiple times, can be described by considering the relationship between the real camera and its virtual camera, reflected by a planar mirror as illustrated in Figure 5. The optical center of the real camera is given as $p_{i}$, and it is assumed that the mirror plane, whose normal vector is $n_{i}$, involves the point $a_{i}$. The optical center of the mirrored virtual camera $p_{i+1}$, which is the reflection of the real camera view on the mirror plane, can be expressed with a 4×4 homogeneous transformation matrix $P_{i}$ as follows:

(1) $$\begin{array}{c} \left(\begin{array}{c} p_{i+1}\\ 1 \end{array}\right)=P_{i}\left(\begin{array}{c} p_{i}\\ 1 \end{array}\right)=\left(\begin{array}{cc} I-2n_{i}n_{i}^{T} & 2\left(n_{i}n_{i}^{T}\right)a_{i}\\ 0 & 1 \end{array}\right)\left(\begin{array}{c} p_{i}\\ 1 \end{array}\right), \end{array}$$

where ***I*** is the 3×3 identity matrix.

#### 3.2.2. Pan-Tilt Mirror System

Using the geometric parameters of the pan-tilt mirror system as defined in Section 3.1.1, the optical center of the virtual camera $p_{pt}$, which is reflected by its pan and tilt mirrors, can be expressed with reflection transformation as follows:

(2) $$\begin{array}{c} \left(\begin{array}{c} p_{pt}\\ 1 \end{array}\right)=P_{2}P_{1}\left(\begin{array}{c} p_{1}\\ 1 \end{array}\right)=P_{2}P_{1}\left(\begin{array}{c} -l\\ 0\\ 0\\ 1 \end{array}\right), \end{array}$$

where

(3) $$\begin{array}{c} P_{1}=\left(\begin{array}{cccc} -\cos2\theta_{1} & \sin2\theta_{1} & \;0 & \;0\\ \sin2\theta_{1} & \cos2\theta_{1} & \;0 & \;0\\ 0 & 0 & \;1 & \;0\\ 0 & 0 & \;0 & \;1 \end{array}\right),\;\;P_{2}=\left(\begin{array}{cccc} 1\; & 0 & 0 & 0\\ 0\; & \cos2\theta_{2} & \sin2\theta_{2} & d(1\;-\;\cos2\theta_{2})\\ 0\; & \sin2\theta_{2} & -\cos2\theta_{2} & -d\sin2\theta_{2}\\ 0\; & 0 & 0 & 1 \end{array}\right). \end{array}$$

Considering $q_{1}={\left(1,0,0\right)}^{T}$, the optical center of the virtual camera $p_{pt}$ and the direction of its optical axis $q_{pt}$ can be derived from Equations (2) and (3), as the following functions of the pan and tilt angles $\theta_{1}$ and $\theta_{2}$,

(4) $$\begin{array}{c} p_{pt}\left(\theta_{1},\theta_{2}\right)=\left(\begin{array}{c} l\cos2\theta_{1}\\ -\left(l\sin2\theta_{1}+d\right)\cos2\theta_{2}+d\\ -\left(l\sin2\theta_{1}+d\right)\sin2\theta_{2} \end{array}\right),\;\;q_{pt}\left(\theta_{1},\theta_{2}\right)=\left(\begin{array}{c} -\cos2\theta_{1}\\ \sin2\theta_{1}\cos2\theta_{2}\\ \sin2\theta_{1}\sin2\theta_{2} \end{array}\right). \end{array}$$

#### 3.2.3. Catadioptric Mirror System

In the catadioptric mirror system, the pan angle of the pan-tilt mirror system determines whether the camera gazes the left view via mirrors 3 and 4 or the right view via mirror 5 and 6.

When the virtual pan-tilt camera gazes the left view, the optical center of the virtual pan-tilt camera after the mirror reflections of the catadioptric mirror system, $p_{L}$, can be expressed by using its geometric parameters described in Section 3.1.2 as follows:

(5) $$\begin{array}{c} \left(\begin{array}{c} p_{L}\\ 1 \end{array}\right)=P_{3}P_{4}\left(\begin{array}{c} p_{pt}\\ 1 \end{array}\right)=P_{3}P_{4}P_{2}P_{1}\left(\begin{array}{c} p_{1}\\ 1 \end{array}\right), \end{array}$$

where

(6) $$\begin{array}{c} P_{3}=\left(\;\;\;\begin{array}{cccc} \cos2\theta_{3} & \;0\; & -\sin2\theta_{3}\; & n\sin2\theta_{3}\\ 0 & \;1\; & 0\; & 0\\ -\sin2\theta_{3} & \;0\; & -\cos2\theta_{3}\; & n(1\;+\;\cos2\theta_{3})\\ 0 & \;0\; & 0\; & 1 \end{array}\;\;\;\;\right),\;P_{4}=\left(\;\;\begin{array}{cccc} -\cos2\theta_{4} & \;0\; & -\sin2\theta_{4}\; & m\sin2\theta_{4}\\ 0 & \;1\; & 0\; & 0\\ -\sin2\theta_{4} & \;0\; & \cos2\theta_{4} & \;\;m(1\;-\;\cos2\theta_{4})\\ 0 & \;0\; & 0\; & 1 \end{array}\;\;\;\;\right). \end{array}$$

Thus, the optical center of the virtual left pan-tilt camera $p_{L}$ and the direction of its optical axis $q_{L}$ can be derived from Equations (4) and (6) as follows:

(7) $$\begin{array}{c} p_{L}\;=\;\left(\;\;\begin{array}{c} -C_{34}l\cos2\theta_{1}\;+\;S_{34}\left(l\sin2\theta_{1}\;+\;d\right)\sin2\theta_{2}\;+\;E\\ -\left(l\sin2\theta_{1}\;+\;d\right)\cos2\theta_{2}\;+\;d\\ S_{34}l\cos2\theta_{1}\;+\;C_{34}\left(l\sin2\theta_{1}\;+\;d\right)\sin2\theta_{2}\;+\;F \end{array}\;\;\right)\;,\;\;q_{L}\;=\;\left(\;\;\begin{array}{c} C_{34}\cos2\theta_{1}-S_{34}\sin2\theta_{1}\sin2\theta_{2}\\ \sin2\theta_{1}\cos2\theta_{2}\\ -S_{34}\cos2\theta_{1}-S_{34}\sin2\theta_{1}\sin2\theta_{2} \end{array}\;\;\right), \end{array}$$

where $C_{34}$, $S_{34}$, *E*, and *F* are constants, which are determined by the parameters of the catadioptric mirror system as follows:

(8) $$\begin{array}{c} C_{34}=\cos2\left(\theta_{3}+\theta_{4}\right),\;\;S_{34}=\sin2\left(\theta_{3}+\theta_{4}\right), \end{array}$$

(9) $$\begin{array}{c} E=m\left(-\sin2\theta_{3}+S_{34}\right)+n\sin2\theta_{3},\;\;F=-m\left(\cos2\theta_{3}-C_{34}\right)+n\left(1+\cos2\theta_{3}\right). \end{array}$$

In a similar manner as the left view via mirrors 3 and 4, the optical center of the virtual pan-tilt camera when the virtual pan-tilt camera gazes the right view via mirrors 5 and 6, $p_{R}$, can be expressed by as follows:

(10) $$\begin{array}{c} \left(\begin{array}{c} p_{R}\\ 1 \end{array}\right)=P_{6}P_{5}\left(\begin{array}{c} p_{pt}\\ 1 \end{array}\right)=P_{6}P_{5}P_{2}P_{1}\left(\begin{array}{c} p_{1}\\ 1 \end{array}\right), \end{array}$$

where

(11) $$\begin{array}{c} P_{5}=\left(\;\;\;\begin{array}{cccc} -\cos2\theta_{5} & \;0\; & \sin2\theta_{5}\; & -m\sin2\theta_{5}\\ 0 & \;1\; & 0\; & 0\\ \sin2\theta_{5} & \;0\; & \cos2\theta_{5}\; & m(1\;-\;\cos2\theta_{5})\\ 0 & \;0\; & 0\; & 1 \end{array}\;\;\;\;\right),\;P_{6}=\left(\;\;\begin{array}{cccc} \cos2\theta_{6} & \;0\; & \sin2\theta_{6}\; & -n\sin2\theta_{6}\\ 0 & \;1\; & 0\; & 0\\ \sin2\theta_{6} & \;0\; & -\cos2\theta_{6}\; & n(1\;+\;\cos2\theta_{6})\\ 0 & \;0\; & 0\; & 1 \end{array}\;\;\;\;\right). \end{array}$$

Considering that the mirrors are symmetrically located with the yz-plane ($\theta_{6}=\theta_{3}$, $\theta_{5}=\theta_{4}$), the optical center of the virtual right pan-tilt camera $p_{R}$ and the direction of its optical axis $q_{R}$ can be derived as follows:

(12) $$\begin{array}{c} p_{R}\;\;=\;\;\left(\;\;\begin{array}{c} -C_{34}l\cos2\theta_{1}\;-\;S_{34}\left(l\sin2\theta_{1}\;+\;d\right)\sin2\theta_{2}\;-\;E\\ -\left(l\sin2\theta_{1}\;+\;d\right)\cos2\theta_{2}\;+\;d\\ -S_{34}l\cos2\theta_{1}\;+\;C_{34}\left(l\sin2\theta_{1}\;+\;d\right)\sin2\theta_{2}\;+\;F \end{array}\;\right)\;\;,\;\;q_{R}\;=\;\left(\begin{array}{c} C_{34}\cos2\theta_{1}+S_{34}\sin2\theta_{1}\sin2\theta_{2}\\ \sin2\theta_{1}\cos2\theta_{2}\\ S_{34}\cos2\theta_{1}-C_{34}\sin2\theta_{1}\sin2\theta_{2} \end{array}\right). \end{array}$$

In our catadioptric stereo tracking, the optical centers and the directions of the optical axes of the virtual left and right pan-tilt cameras are controlled so that the apparent target positions on their image sensor planes, $u_{L}=\left(u_{L},v_{L}\right)$ and $u_{R}=\left(u_{R},v_{R}\right)$, are tracked in the view fields of the virtual left and right pan-tilt cameras, respectively.

## 4. Catadioptric Stereo Tracking System

### 4.1. System Configuration

**Figure 6:**
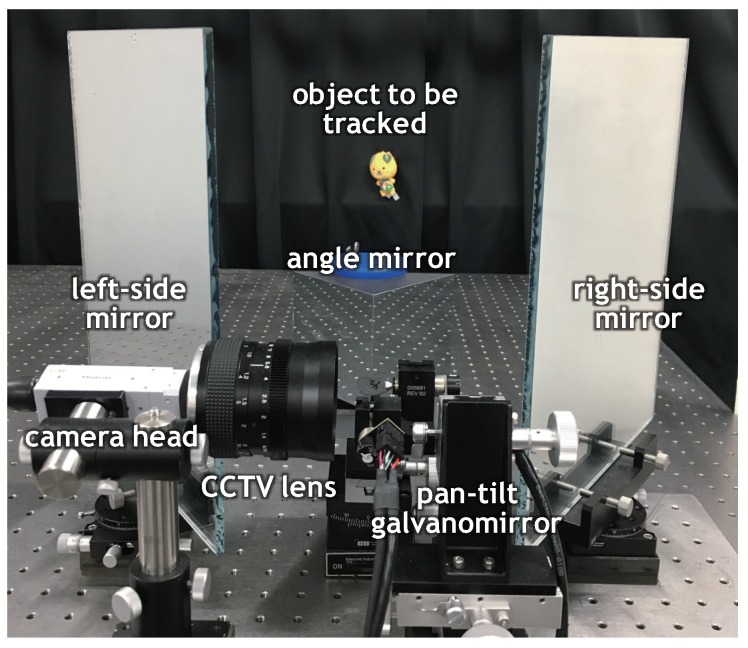
Overview of catadioptric stereo tracking system.

We developed a catadioptric stereo tracking system, designed for multithreaded gaze control for switching left and right viewpoints frame-by-frame to capture a pair of stereo images for fast-moving objects in 3D scenes. The system consists of a high-speed vision platform (IDP Express) [78], a pan-tilt galvano-mirror (6210H, Cambridge Technology Inc., Bedford, MA, USA), a right-angle mirror and two flat mirrors on the left and right sides, and a personal computer (PC) (Windows 7 Enterprise 64-bit OS (Microsoft, Redmond, WA, USA); ASUS P6T7 WS SuperComputer motherboard (ASUS, Taiwan, China); Intel Core (TM) i7 3.20-GHz CPU, 6 GB memory (Intel, Santa Clara, CA, USA)). A D/A board (PEX-340416, Interface Inc., Hiroshima, Japan) is used to send control signals to the galvano-mirror and an A/D board (PEX-321216, Interface Inc., Hiroshima, Japan) is used to collect the sensor signals of the pan and tilt angles of the galvano-mirror. Figure 6 provides an overview of our developed catadioptric stereo tracking system.

The high-speed vision platform IDP Express (R2000, Photron, Tokyo, Japan) consists of a compact camera head and an FPGA image processing board (IDP Express board). The camera head has a Complementary Metal Oxide Semiconductor (CMOS) image sensor (C-MOS, Photron, Tokyo, Japan) of 512×512 pixels, with a sensor size and pixel size of 5.12 × 5.12 mm and 10 × 10 μm, respectively. The camera head can capture 8-bit RGB (Red, Green, Blue) images of 512 × 512 pixels at 2000 fps with a Bayer filter on its image sensor. A f= 50 mm CCTV (Closed Circuit Television) lens is attached to the camera head. The IDP Express board was designed for high-speed video processing and recording, and image processing algorithms can be hardware-implemented on the FPGA (Xilinx XC3S5000, Xilinx Inc., San Jose, CA, USA). The 8-bit color 512 × 512 images and processed results are simultaneously transferred at 2000 fps from the IDP Express board via the Peripheral Component Interconnect (PCI)-e 2.0 × 16 bus to the allocated memory in the PC.

**Figure 7:**
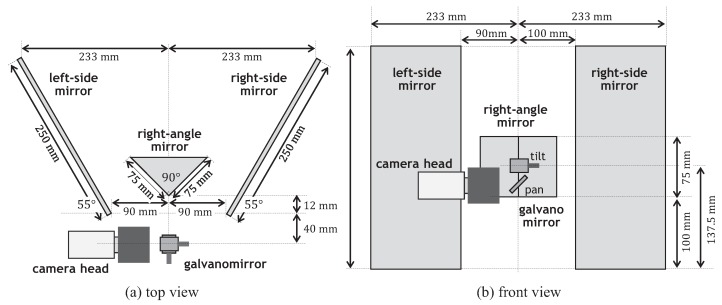
Arrangement of mirror system: (**a**) top view; (**b**) front view.

The galvano-mirror can control two-degrees-of-freedom (DOF) gazes using pan and tilt mirrors, whose sizes are 10.2 mm${}^{2}$ and 17.5 mm${}^{2}$, respectively. By applying a voltage signal via the D/A board, the angles of the pan and tilt mirrors are movable in the range of −10 to 10 degrees, and they can be controlled within 1 ms in the range of 10 degrees. The pan mirror of the galvano-mirror was installed 25 mm in front of the CCTV lens, and the tilt mirror was installed 10 mm in front of the pan mirror. A right-angle mirror, whose lengths of the hypotenuse and legs are 106.1 mm and 75.0 mm, respectively, and two 250 × 310 mm flat mirrors were symmetrically located in front of the galvano-mirror. The catadioptric mirror system was set for short-distance stereo measurement to verify the performance of our catadioptric stereo tracking system in a desktop environment. This enabled us to easily manage the lighting condition to cope with insufficient incident light, owing to the small pan-tilt mirror, and to quantitatively moving at apparently high speeds in images using a linear slider. The configuration of a catadioptric mirror system consisting of these mirrors is illustrated in Figure 7. The right-angle mirror was installed in front of the tilt mirror of the galvano-mirror, so that the optical axis of the CCTV lens, which was reflected on the galvano-mirror, comes to the middle point of the right-angled side of the right-angle mirror when the pan and tilt angles of the galvano-mirror were zero, corresponding to the center angles in their movable ranges. On the left and right sides, two flat mirrors were vertically installed with an angle of 55 degrees.

**Figure 8:**
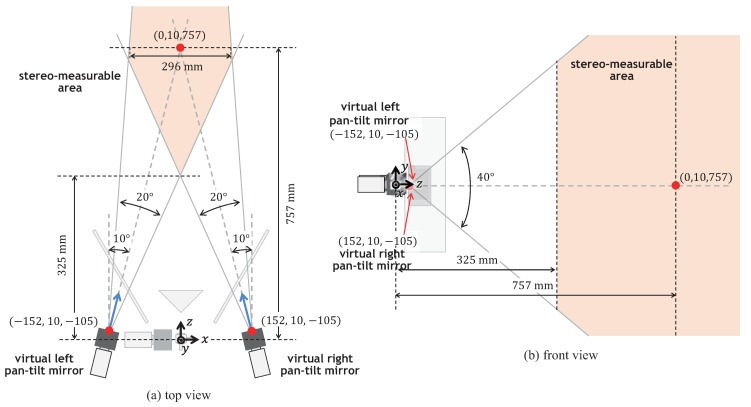
Virtual pan-tilt cameras and stereo-measurable area: (**a**) top view; (**b**) front view.

The right angle mirror and the two side mirrors can cover the view angle of the camera view completely when the pan and tilt angles of the galvano-mirror were in the range of −10 to 10 degrees, whereas the camera view involved the left-side and right-side views, which were split by the right-angle mirror, when the pan angle of the galvano-mirror was around zero; the camera view only involved the left-view image via the left-side mirror or the right-view image via the right-side mirror, when the pan angle was in the range of −10 to −2 degrees or in the range of 2 to 10 degrees, respectively. It is assumed that the reference positions of the virtual left and right pan-tilt cameras were set when the pan and tilt angles of the galvano-mirror were −5 and 0 degrees, and 5 and 0 degrees, respectively. Figure 8 illustrates the locations of the virtual left and right pan-tilt cameras. The optical centers and the normal direction vectors of the optical axes of the virtual left and right cameras at their reference positions were (−152mm,10mm,−105mm) and (0.174,0.000,0.985), and (152mm,10mm,−105mm) and (−0.174,0.000,0.985), respectively; the xyz-coordinate system was set so that its origin was set to the center of the pan mirror of the galvano-mirror as illustrated in Figure 8. The virtual left and right pan-tilt cameras can change their virtual pan and tilt angles around their reference positions in the range of −10 to 10 degrees and in the range of −20 to 20 degrees, respectively, which corresponded to twice of the movable ranges of the pan and tilt angles of the galvano-mirror for each virtual pan-tilt camera, whereas the view angle of the camera view was 8.28 degrees in both the pan and tilt directions, respectively, which were determined by the focal distance f= 50 mm of the CCTV lens and the 5.12 × 5.12 mm size of the image sensor.

The stereo-measurable area where a pair of left-view and right-view images can be captured is also illustrated in Figure 8. The stereo-measurable area is 296 × 657 mm on a vertical plane 757 mm in front of the tilt mirror of the galvano-mirror, on which the optical axes of two virtual pan-tilt cameras at their reference positions were crossed, whereas the left-view and right-view images of 512 × 512 pixels corresponded to 94 × 94 mm on the vertical plane. When the virtual left and right pan-tilt cameras were at their reference positions, the error in stereo measurement of a nonmoving object 757 mm in front of the tilt mirror of the galvano-mirror, was ±0.10 mm in the *x*-direction, ±0.05 mm in the *y*-direction, and ±0.2 mm in the *z*-direction, respectively.

### 4.2. Implemented Algorithm

**Figure 9:**
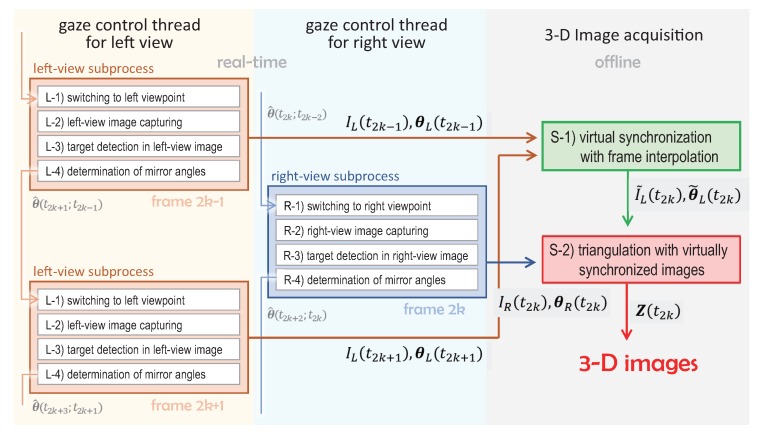
Flowchart of the implemented algorithm.

Assuming that the target scene to be tracked is textured with a specific color, we implement an algorithm to calculate the 3D images using the virtual left and right-view images captured through the ultrafast pan-tilt mirror system: (1) a stereo tracking process with multithread gaze control; and (2) a 3D image estimation process with virtually synchronized images. In this study, the stereo tracking process (1) is executed in real time for mechanical tracking control to keep the target object in view of the virtual left and right pan-tilt cameras with visual feedback, while the 3D image estimation process (2) is executed offline using the left and right-view images, and the pan and tilt angles of the virtual pan-tilt mirror systems, which are being logged during the stereo tracking. Figure 9 shows the flowchart of the algorithm.

#### 4.2.1. Stereo Tracking Process with Multithread Gaze Control

In the stereo tracking process, the left and right-view subprocesses for multithread gaze control are alternatively switched at a small interval of Δt. The left-view subprocess works for $t_{2k-1}-\tau_{m}\leq t<t_{2k}-\tau_{m}$, and that of the right view works for $t_{2k}-\tau_{m}\leq t<t_{2k+1}-\tau_{m}$ as the time-division thread executes with a temporal granularity of Δt. $t_{k}=t_{0}+k\Delta t$ (*k*: integer) indicates the image-capturing time of the high-speed vision system, and $\tau_{m}$ is the settling time in controlling the mirror angles of the pan-tilt mirror system.

[Left-view subprocess]

(L-1) Switching to the left viewpoint

For time $t_{2k-1}-\tau_{m}$ to $t_{2k-1}$, the pan and tilt angles of the pan-tilt mirror system are controlled to within their desired values $\hat{\theta }\left(t_{2k-1};t_{2k-3}\right)=\left({\hat{\theta }}_{1}\left(t_{2k-1};t_{2k-3}\right),{\hat{\theta }}_{2}\left(t_{2k-1};t_{2k-3}\right)\right)$ at time $t_{2k-1}$, which is estimated at time $t_{2k-3}$ when capturing the left-view image in the previous frame.

(L-2) Left-view image capturing

The left-view image $I(t_{2k-1})$ is captured at time $t_{2k-1}$; I(t) indicates the input image of the high-speed vision system at time *t*.

(L-3) Target detection in left-view image

The target object with a specific color is localized by detecting its center position u(t)=(u(t),v(t)) in the image I(t) at time *t*. Assuming that the color of the target object to be tracked is different from its background color in this study, $u(t_{2k-1})$ is calculated as a moment centroid of a binary image $C\left(t_{2k-1}\right)=C\left(u,v,t_{2k-1}\right)$ for the target object as follows:

(13) $$\begin{array}{c} \;u\left(t_{2k-1}\right)=\left(M_{u}/M_{0},M_{v}/M_{0}\right), \end{array}$$

(14) $$\begin{array}{c} \;M_{0}=\sum_{u,v}C\left(u,v,t_{2k-1}\right),\;\;M_{u}=\sum_{u,v}uC\left(u,v,t_{2k-1}\right),\;\;M_{v}=\sum_{u,v}vC\left(u,v,t_{2k-1}\right), \end{array}$$

where the binary image C(t) is obtained at time *t* by setting a threshold for the HSV (Hue, Saturation, Value) images as follows:

(15) $$\begin{array}{c} C\left(t\right)=\left\{\begin{array}{cc} 1, & (H_{l}\leq H<H_{h},S>S_{l},V>V_{l}),\\ 0, & (\mathrm{orherwise}), \end{array}\right. \end{array}$$

where *H*, *S*, and *V* are the hue, saturation, and value images of I(t), respectively. $H_{l}$, $H_{h}$, $S_{l}$, and $V_{l}$ are parameters for HSV color thresholding.

(L-4) Determination of mirror angles at the next left-view frame

Assuming that the *u*- and *v*-directions in the image correspond to the pan and tilt directions of the pan-tilt mirror system, respectively, the pan and tilt angles at time $t_{2k+1}$ when capturing the left-view image at the next frame, are determined so as to reduce the error between the position of the target object and its desired position $u_{L}^{d}$ in the left-view image with proportional control as follows:

(16) $$\begin{array}{c} \hat{\theta }\left(t_{2k+1};t_{2k-1}\right)=-K\left(u\left(t_{2k-1}\right)-u_{L}^{d}\right)+\theta \left(t_{2k-1}\right), \end{array}$$

where $\theta \left(t\right)=(\theta_{1}\left(t\right),\theta_{2}\left(t\right))$ is collectively the measured values of the pan and tilt angles at time *t*, and *K* is the gain parameter for tracking control.

[Right-view subprocess]

(R-1) Switching to right viewpoint

For time $t_{2k}-\tau_{m}$ to $t_{2k}$, the pan and tilt angles are controlled to $\hat{\theta }\left(t_{2k};t_{2k-2}\right)$, which is estimated at time $t_{2k-2}$ when capturing the right-view image in the next frame.

(R-2) Right-view image capturing

The right-view image $I(t_{2k})$ is captured at time $t_{2k}$.

(R-3) Target detection in right-view image

$u\left(t_{2k}\right)=\left(u\left(t_{2k}\right),v\left(t_{2k}\right)\right)$ is obtained as the center position of the target object in the right-view image at time $t_{2k}$, by calculating a moment centroid of $C(t_{2k})$, which is a sub-image $I(t_{2k})$ of the right-view image, constrained by a color threshold at time $t_{2k}$, in a similar manner as that described in L-3.

(R-4) Determination of mirror angles in the next right-view frame

Similarly, with the process described in L-4, the pan and tilt angles at time $t_{2k+2}$ when capturing the right-view image in the next frame are determined as follows:

(17) $$\begin{array}{c} \hat{\theta }\left(t_{2k+2};t_{2k}\right)=-K\left(u\left(t_{2k}\right)-u_{R}^{d}\right)+\theta \left(t_{2k}\right), \end{array}$$

where $u_{R}^{d}$ is the desired position of the target object in the right-view image.

The input images and the mirror angles captured in the stereo tracking process are stored as the left-view images $I_{L}\left(t_{2k-1}\right)=I\left(t_{2k-1}\right)$ and the pan and tilt angles $\theta_{L}\left(t_{2k-1}\right)=\theta \left(t_{2k-1}\right)$ at time $t_{2k-1}$, for the virtual left pan-tilt camera at the odd-numbered frame, and the right-view images $I_{R}\left(t_{2k}\right)=I\left(t_{2k}\right)$ and the pan and tilt angles $\theta_{R}\left(t_{2k}\right)=\theta \left(t_{2k}\right)$ at time $t_{2k}$ for the virtual right pan-tilt camera at the even-numbered frame.

#### 4.2.2. 3D Image Estimation with Virtually Synchronized Images

Left and right-view images in catadioptric stereo tracking are captured at different timings, and the synchronization errors in stereo measurement increase as the target object’s movement increases. To reduce such errors, this study introduces a frame interpolation technique for virtual synchronization between virtual left and right pan-tilt cameras, and 3D images are estimated with stereo processing for the virtually synchronized left and right-view images. Frame interpolation is a well-known video processing technique in which intermediate frames are generated between existing frames by means of interpolation using space-time tracking [79,80,81,82], view morphing [83,84,85], and optical flow [86,87]; it has been used for many applications, such as frame rate conversion, temporal upsampling for fluid slow motion video, and image morphing.

(S-1) Virtual Synchronization with Frame Interpolation

Considering the right-view image $I_{R}\left(t_{2k}\right)$ captured at time $t_{2k}$ as the standard image for virtual synchronization, the left-view image virtually synchronized at time $t_{2k}$, ${\tilde{I}}_{L}\left(t_{2k}\right)$, is estimated with frame interpolation using the two temporally neighboring left-view images $I_{L}\left(t_{2k-1}\right)$ at time $t_{2k-1}$ and $I_{L}\left(t_{2k+1}\right)$ at time $t_{2k+1}$ as follows:

(18) $$\begin{array}{c} {\tilde{I}}_{L}\left(t_{2k}\right)=f_{FI}\left(I_{L}\left(t_{2k-1}\right),I_{L}\left(t_{2k+1}\right)\right), \end{array}$$

where $f_{FI}\left(I_{1},I_{2}\right)$ indicates the frame interpolation function using two images $I_{1}$ and $I_{2}$. We used Meyer’s phase-based method [88] as the frame interpolation technique in this study.

In a similar manner, the pan and tilt angles of the left pan-tilt camera are virtually synchronized with those of the right pan-tilt camera at time $t_{2k}$, ${\tilde{\theta }}_{L}\left(t_{2k}\right)$, are also estimated using the temporally neighboring mirror angles $\theta_{L}\left(t_{2k-1}\right)$ at time $t_{2k-1}$ and $\theta_{L}\left(t_{2k+1}\right)$ at time $t_{2k+1}$ as follows:

(19) $$\begin{array}{c} {\tilde{\theta }}_{L}\left(t_{2k}\right)=\frac{1}{2}\left(\theta_{L}\left(t_{2k-1}\right)+\theta_{L}\left(t_{2k+1}\right)\right), \end{array}$$

where it is assumed that the mirror angles of the virtual left pan-tilt camera vary linearly for the interval 2Δt during time $t_{2k-1}$ and $t_{2k+1}$.

(S-2) Triangulation Using Virtually Synchronized Images

The virtually synchronized left and right-view images at time $t_{2k}$, ${\tilde{I}}_{L}\left(t_{2k}\right)$ and $I_{R}\left(t_{2k}\right)$, are used to compute the 3D image of the tracked object in a similar way as those in the standard stereo methodologies for multiple synchronized cameras. Assuming that the camera parameters of the virtual pan-tilt camera at arbitrary pan and tilt angles θ are initially given as the 3 × 4 camera calibration matrix P(θ), the 3D image $Z(t_{2k})$ can be estimated at time $t_{2k}$ as a disparity map as follows:

(20) $$\begin{array}{c} Z\left(t_{2k}\right)=f_{dm}({\tilde{I}}_{L}\left(t_{2k}\right),I_{R}\left(t_{2k}\right);P\left({\tilde{\theta }}_{L}\left(t_{2k}\right)\right),P\left(\theta_{R}\left(t_{2k}\right)\right), \end{array}$$

where $f_{dm}\left(I_{L},I_{R};P_{L},P_{R}\right)$ indicates the function of stereo matching using a pair of left and right-view images, $I_{L}$ and $I_{R}$, when the 3 × 4 camera calibration matrices of the left- and right cameras are given as $P_{L}$ and $P_{R}$, respectively. We used the rSGM method [89] as the stereo matching algorithm in this study.

### 4.3. Specifications

In the stereo tracking process, the viewpoint switching steps (L-1, R-1) require $\tau_{m}=$ 1 ms for the settling time in mirror control, and the image capturing steps (L-2, R-2) require 0.266 ms. The execution time of the target detection steps (L-3,4, R-3,4) is within 0.001 ms, which is accelerated by hardware-implementing the target detection circuit by setting a threshold for the HSV color in Equations (13)–(15) on the user-specific FPGA of the IDP Express board. Here, the mirrors of the pan-tilt system should be in a state of rest for motion blur reduction in the captured images; the camera exposure in the image capturing steps cannot be executed during the viewpoint switching steps, whereas the target detection steps can be executed in parallel with the next viewpoint switching steps. Thus, the switching time of the left and right-view images is set to Δt = 2 ms, so that the settling time in mirror control is $\tau_{m}=$ 1 ms and the exposure time is 1 ms. We have confirmed that the stereo tracking process could capture and process a pair of the left- and right-view 8-bit color 512 × 512 images in real time at 250 fps using a single camera operating at 500 fps.

In this study, the 3D image estimation is executed offline because the computation it requires is too heavy to process 512 × 512 images in real time at 250 fps on our catadioptric tracking system; the execution time for virtual synchronization with frame interpolation is 54 s, and that for 3D image estimation is approximately 906.3 ms. The 3D image estimation with the rSGM method is too time-consuming to conduct real-time applications such as Simultaneous Localization and Mapping (SLAM) problems and large scale mapping, whereas the current setup can be used for precise 3D digital archiving/video logging to estimate the 3D images of small objects and creatures fast-moving in the wide area. Our catadioptric stereo tracking system functions as an active stereo system, and its complexity in calibration is similar to those of standard active stereo systems, where there is a trade-off between the complexity and accuracy of the calibration. In this study, focusing on calibration accuracy, the initial look-up-table camera-calibration matrices $P_{lut}\left(\theta_{ij}\right)$ (i=1,⋯,52,j=1,⋯,31) for 3D image estimation are determined at 52 × 31 different mirror angles by applying Zhang’s calibration method [46] to the captured images of a calibration checkered board at each mirror angle; the pan angle in the range of −10 to −5 degrees and 5 to 10 degrees at intervals of 0.2 degrees, and the tilt angle in the range of −3 to 3 degrees at intervals of 0.2 degrees. In this study, the camera calibration matrix P(θ) at the mirror angle θ is linearly interpolated with the look-up-table matrices $P_{lut}$ at the four nearest neighbor mirror angles around θ; it can be measured accurately by the angular sensor of the galvano-mirror system at all times, including when the mirror angle is not controlled perfectly to its desired value. Here, it is noted that the offline 3D image estimation that involves heavy computation and the complexity in the camera calibration are the common issues in standard active stereo systems using multiple PTZ cameras, as well as in our catadioptric stereo tracking system.

## 5. Experiments

### 5.1. 3D Shapes of Stationary Objects

**Figure 10:**
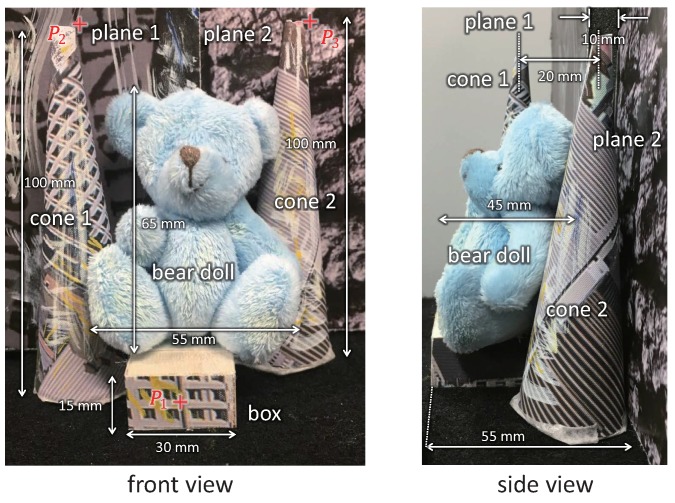
3D scene to be observed.

First, we measured the 3D shapes for the stationary objects at different depths. Figure 10 shows the target objects to be measured; a cyan-colored bear doll of 55 × 65 × 45 mm size sitting on a box of 30 × 15 × 55 mm size, two 100 mm-height textured cones, and two differently textured background planes with a depth gap of 10 mm. Except for the bear doll to be tracked, all the objects are black-and-white textured. They were fixed as a rigid-body scene and can move in the *x*- or *z*-directions by a linear slider. In the experiment, the cyan-colored regions in the virtual left and right-view images were mechanically tracked at $u_{L}^{d}=\left(310,255\right)$ and $u_{R}^{d}=\left(200,255\right)$, respectively; the parameters of the cyan-colored region extraction for 8-bit HSV images were set to $H_{l}$ = 85, $H_{h}$ = 110, $S_{l}$ = 20, and $V_{l}$ = 80. The gain parameter for mirror control was set to *K* = 0.01.

**Figure 11:**
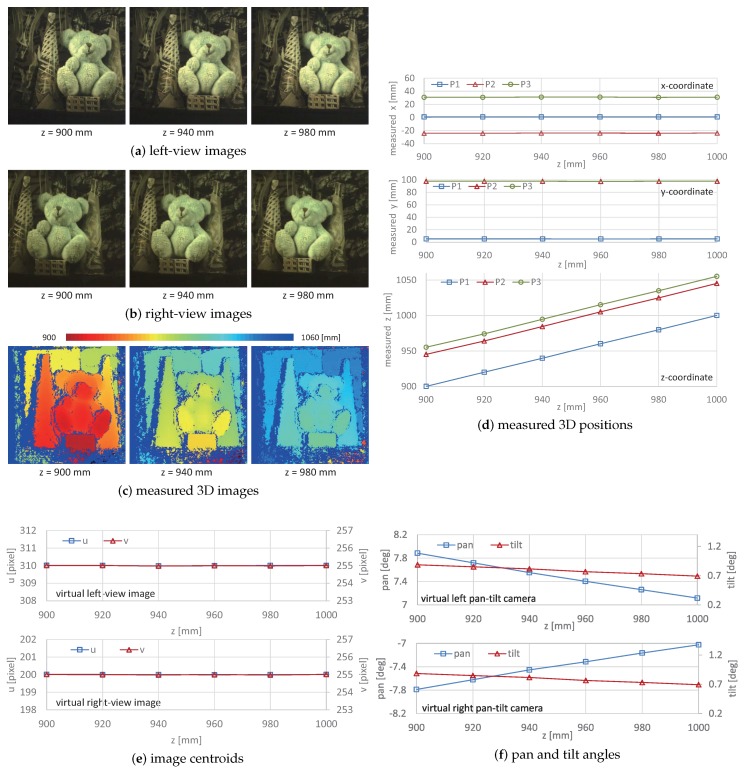
Measured 3D images and positions, image centroids, and pan and tilt angles of virtual left and right pan-tilt cameras for stationary 3D scenes at different depths: (**a**) left-view images; (**b**) right-view images; (**c**) measured 3D images; (**d**) measured 3D positions; (**e**) image centroids; (**f**) pan and tilt angles.

Figure 11 shows (a) the left-view images; (b) the right-view images; and (c) the measured 3D images when the distance between the point $P_{1}$ on the box under the doll and the system varied along a straight line of x= 0.9 mm and y= 5.3 mm at z= 900.0, 940.0, and 980.0 mm. (d) the xyz coordinate values of the points $P_{1}$, $P_{2}$, and $P_{3}$, (e) the xy-centroids, and (f) the pan and tilt angles of the virtual left and right pan-tilt cameras when the target scene was located at different depths from z= 900 to 1000 mm at intervals of 20 mm. The point $P_{1}$ is located at the center of the front surface of the box, and the points $P_{2}$ and $P_{3}$ are located at the left and right-side background planes, respectively; their actual xyz-coordinate values were $P_{1}$ (0.9 mm, 5.3 mm, 900.0 mm), $P_{2}$ (−24.0 mm, 97.8 mm, 945.0 mm), and $P_{3}$ (31.0 mm, 97.8 mm, 955.0 mm). In Figure 11c, the 3D shapes of the bear doll, the box, the two cones, and the background planes with a 10 mm depth gap are accurately measured, and they were translated in the *z*-direction, corresponding to the distance from the system. The xyz-coordinate values almost match the actual coordinate values, and the measurement errors were always within 1.0 mm. The pan angles in both the virtual left and right pan-tilt cameras slightly decreased as the distance between the target object and the system became larger, whereas the xy centroids in the left and right-view images were always held to within (310,255) and (200,255), respectively; the tracking errors were always within 0.1 pixel. Thus, our catadioptric stereo tracking system can correctly measure the 3D shapes of the stationary target objects.

**Figure 12:**
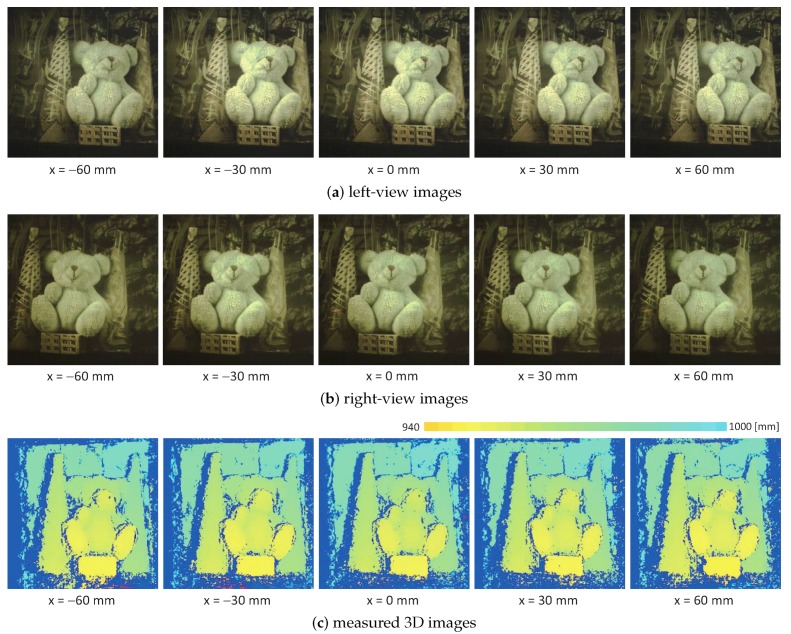
Measured 3D images for stationary 3D scenes at different *x*-coordinates when the target object was mechanically tracked in both the left- and right-view images: (**a**) left-view images; (**b**) right-view images; (**c**) measured 3D images.

**Figure 13:**
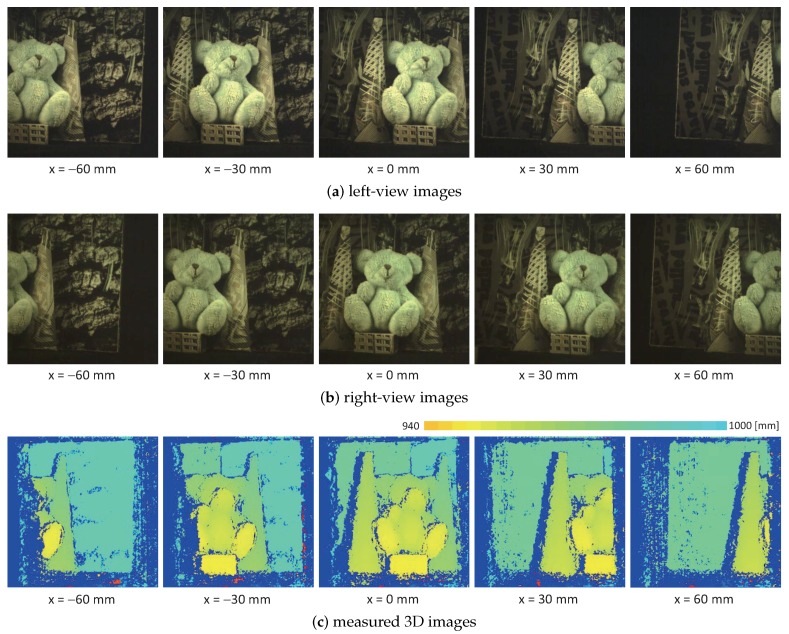
Measured 3D images for stationary 3D scenes at different *x*-coordinates when the mirror angles of the virtual left and right pan-tilt cameras were fixed without tracking: (**a**) left-view images; (**b**) right-view images; (**c**) measured 3D images.

Next, we measured the 3D images of the target object when the distance between the point $P_{1}$ and the system varied along a straight line of y= 5.3 mm and z= 940.0 mm at x= −60.0, −30.0, 0.0, 30.0, and 60.0 mm. Figure 12 shows the (a) left-view images; (b) right-view images; and (c) measured 3D images when the target object was mechanically tracked in both the left- and right-view images with multithread gaze control. For the same scenes at different locations, Figure 13 shows the experimental results when the mirror angles of the virtual left and right pan-tilt cameras were fixed without tracking; the target object located at x= 0.0 mm was observed at (310,255) and (200,255) in the left- and right-view images, respectively. In Figure 13, the target object located at x=−60.0 and 60.0 mm was almost out of the measurable range in the stereo measurement without tracking, whereas the 3D images of the target object were observable continuously in the stereo measurement with tracking, as illustrated in Figure 12. Thus, our catadioptric stereo tracking system can expand the measurable area without decreasing resolution by mechanically tracking the target object in both the left- and right-view images, even for the short-distance experiments detailed in this subsection.

### 5.2. 3D Shape of Moving Objects

Next, the 3D images of a moving scene at different velocities were measured; the same scene used in the previous subsection was conveyed in the *x*- and *z*-directions by a linear slider. The virtual left and right-view images were tracked by setting the same parameters used in the previous subsection.

**Figure 14:**
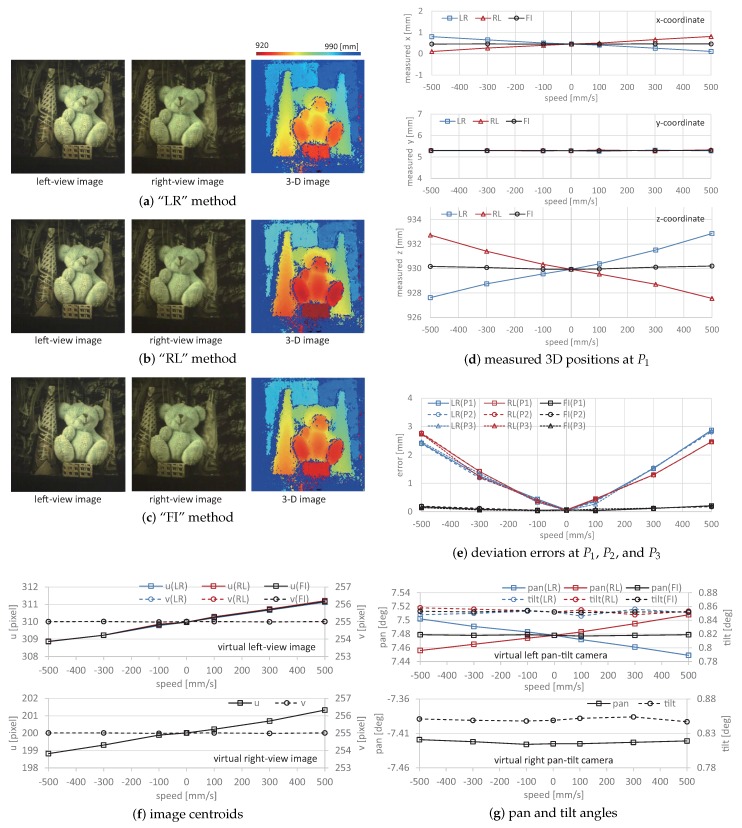
Left- and right-view images, measured 3D images and positions, deviation errors, image centroids, and pan and tilt angles of virtual left and right pan-tilt cameras when moving at 500 mm/s in the *x*-direction: (**a**) “LR” method; (**b**) “RL” method; (**c**) “FI” method; (**d**) measured 3D positions at $P_{1}$; (**e**) deviation errors at $P_{1}$ , $P_{2}$ , and $P_{3}$; (**f**) image centroids; (**g**) pan and tilt angles.

Figure 14a–c shows the left and right-view images, and the measured 3D images when the point $P_{1}$ on the box was around (x,y,z)= (0.5 mm, 5.3 mm, 930.0 mm); the target scene moved at 500 mm/s in the *x*-direction. The 3D images $Z(t_{2k})$ measured by using the virtually synchronized left and right-view images ((c) the “FI” method) were illustrated as well as $Z_{-}\left(t_{2k}\right)$ and $Z_{+}\left(t_{2k}\right)$, which were measured by using the left-view image and the right-view one with a 2 ms delay ((a) the “LR” method), and the right-view image and the left-view one with a 2 ms delay ((b) the `RL” method), respectively:

(21) $$\begin{array}{c} Z_{-}\left(t_{2k}\right)=f_{dm}(I_{L}\left(t_{2k-1}\right),I_{R}\left(t_{2k}\right);P\left(\theta_{L}\left(t_{2k-1}\right)\right),P\left(\theta_{R}\left(t_{2k}\right)\right), \end{array}$$

(22) $$\begin{array}{c} Z_{+}\left(t_{2k}\right)=f_{dm}(I_{L}\left(t_{2k+1}\right),I_{R}\left(t_{2k}\right);P\left(\theta_{L}\left(t_{2k+1}\right)\right),P\left(\theta_{R}\left(t_{2k}\right)\right). \end{array}$$

**Figure 15:**
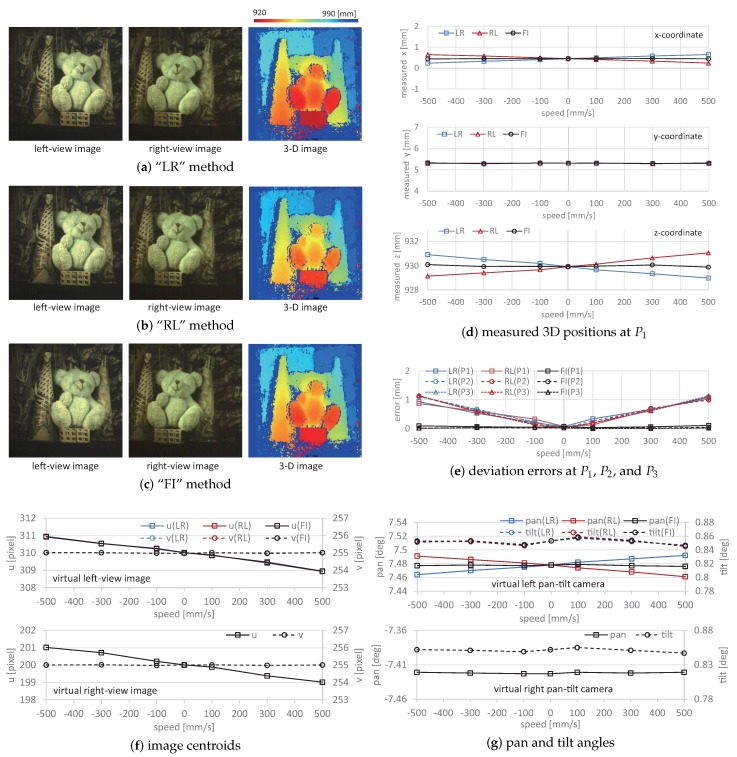
Left- and right-view images, measured 3D images and positions, deviation errors, image centroids, and pan and tilt angles of virtual left and right pan-tilt cameras when moving at 500 mm/s in the *z*-direction: (**a**) “LR” method; (**b**) “RL” method; (**c**) “FI” method; (**d**) measured 3D positions at $P_{1}$; (**e**) deviation errors at $P_{1}$ , $P_{2}$ , and $P_{3}$; (**f**) image centroids; (**g**) pan and tilt angles.

Figure 14d–f shows the measured 3D positions at the point $P_{1}$, the deviation errors from the actual 3D positions at the points $P_{1}$, $P_{2}$, and $P_{3}$, the image centroids, and the pan and tilt angles of the virtual left and right pan-tilt cameras when the target scene moved at different speeds from −500, −300, −100, 0, 100, 300, and 500 mm/s in the *x*-direction; the actual positions were $P_{1}$(0.5 mm, 5.3 mm, 930.0 mm), $P_{2}$(−24.5 mm, 97.8 mm, 975.0 mm), and $P_{3}$(30.5 mm, 97.8 mm, 985.0 mm). The 3D positions and errors measured by the “FI” method were compared with those measured by the “LR” and “RL” methods. The pan and tilt angles and image centroids of the virtual right pan-tilt camera were common in all of the measurements, whereas those of the virtual left one differed according to whether virtual synchronization was active. Similarly, Figure 15 shows (a)–(c) the left and right-view images, and the measured 3D images when the target scene moved at 500 mm/s in the *z*-direction; (d) the measured 3D positions at the point $P_{1}$; (e) the deviation errors from the actual 3D positions at the points $P_{1}$, $P_{2}$, and $P_{3}$; (f) the image centroids; and (g) the pan and tilt angles of the virtual left and right pan-tilt cameras when the target scene moved at different speeds in the *z*-direction.

The 3D positions measured by the “FI” method were almost constant when the target scene moved at different speeds in the *x*- and *z*-directions, whereas the deviations of the *y*- and *z*-coordinate values measured by “LR” and “RL” methods from those measured when the target scene had no motion increased with the amplitude of the target’s speed. The deviation errors at the point $P_{1}$ when the target scene moved at 500 mm/s in the *x*-direction were 2.87. 2.46, and 0.20 mm, respectively, and those when the target scene moved at 500 mm/s in the *z*-direction, were 1.03, 1.08, and 0.11 mm, respectively; the deviation errors in the “FI” measurement were approximately 1/10 of those in the “LR” and “RL” measurements. A similar tendency was observed in the deviation errors at the points $P_{2}$ and $P_{3}$. In our system, the target object was always tracked to the desired positions in the left and right-view images by controlling the pan and tilt angles of the virtual left and right pan-tilt cameras.

In Figure 14f and Figure 15f, the image centroids in the left and right-view images slightly deviated from their desired positions in proportion to the target’s speed, which was dependent on the operational limit of the pan-tilt mirror system. This tendency was similar in the “FI”, “LR”, and “RL” measurements; the deviations of the image centroids in the left and right-view images when the target scene moved at 500 mm/s in the *x*- and *z*-directions were 1.2 and 1.3 pixels and 1.0 and 1.0 pixels, respectively. In Figure 14 and Figure 15, the left-view images in the “FI”, “LR”, and “RL” measurements were similar, and the differences of the apparent positions of $P_{1}$, $P_{2}$, and $P_{3}$ in all the measurements were within one pixel when the target scene moved at 500 mm/s in the *x*- and *z*-directions. This is because the target scene moved together with its background objects as a rigid body, and the left-view images were not so largely varied by tracking the color-patterned object to its desired position in the images.

In Figure 14g and Figure 15g, the pan angle in the left pan-tilt camera in the “FI” measurement when the target scene moved at different speeds in the *x*- and *z*-directions was 7.478 degrees, the same as that when the target scene had no motion, whereas those in the “LR” and “RL” measurements deviated in proportion to the target’s speed; those in the “LR” and “RL” measurements when 500 mm/s in the *x*- and *z*-directions were 7.449 and 7.508 degrees, and 7.492 and 7.461 degrees, respectively. The tilt angle of the left pan-tilt camera and the pan and tilt angles of the right pan-tilt camera were almost similar at different speeds in the *x*- and *z*-directions. The measurement errors in the “LR” and “RL” measurements, in which the virtual left pan-tilt camera was not virtually synchronized with the right one, were mainly caused by these deviations in the pan angle of the left pan-tilt camera, whereas the left-view images were almost similar in the “LR”, “RL”, and “FI” measurements. This indicates that the 3D images measured by the “FI” method were accurately estimated as the same information as when the target scene moved at different speeds in the *x*- and *z*-directions because the synchronization errors in stereo computation were remarkably reduced by synchronizing virtual pan-tilt cameras with frame interpolation. In contrast, the 2-ms interval between the left- and right-view images was not sufficiently large, and the deviation errors were not serious even when the object speed in the experiment was 500 mm/s. Virtual synchronization between left- and right-view images is more effective when a large galvano-mirror system is used for viewpoint-switching with sufficient incident light. This is because the synchronization errors increase when the switching time between the left- and right-view images increases according to the mirror size.

### 5.3. Dancing Doll in 3D Space

**Figure 16:**
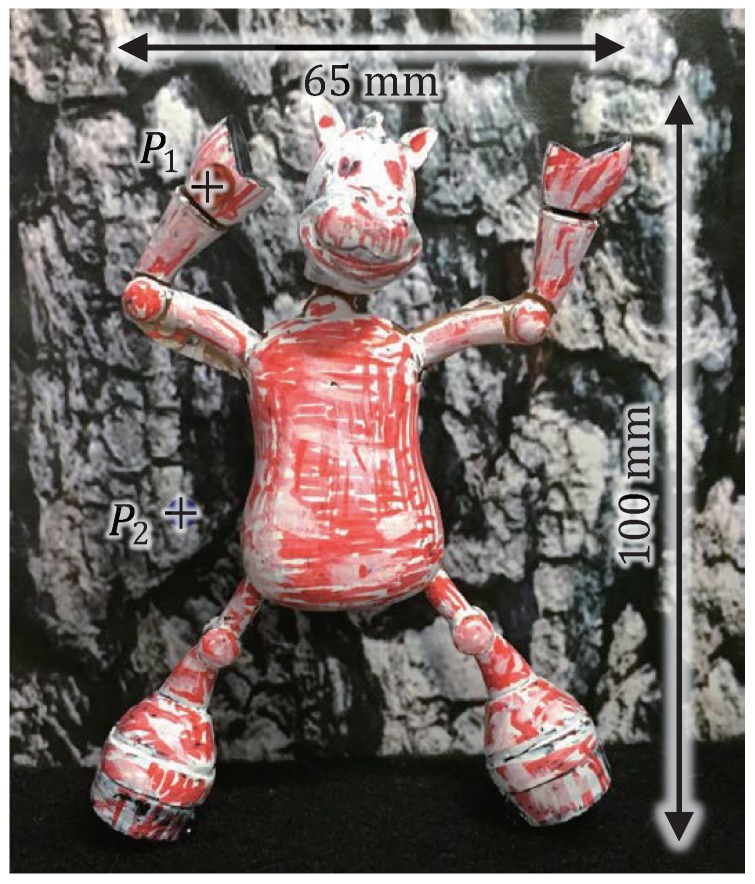
Dancing horse doll to be observed.

Finally, we measured the 3D shapes of a dancing horse doll of size 63 × 25 × 100 mm as illustrated in Figure 16. The doll was dancing 40 mm in front of a background plane with black and white patterns. The surface of doll was textured with red color. The virtual left and right-view images were tracked by setting the same parameters used in the previous subsection, excluding the parameters for red-colored region extraction, $H_{l}$ = 0, $H_{h}$ = 65, $S_{l}$ = 61, and $V_{l}$ = 115. The doll and the background plane was moved together at 100 mm/s in the *z*-direction from *z* = 900 to 1000 mm by the linear slider while the doll was dancing with shaking its body and legs at a frequency of approximately 2.5 Hz.

**Figure 17:**
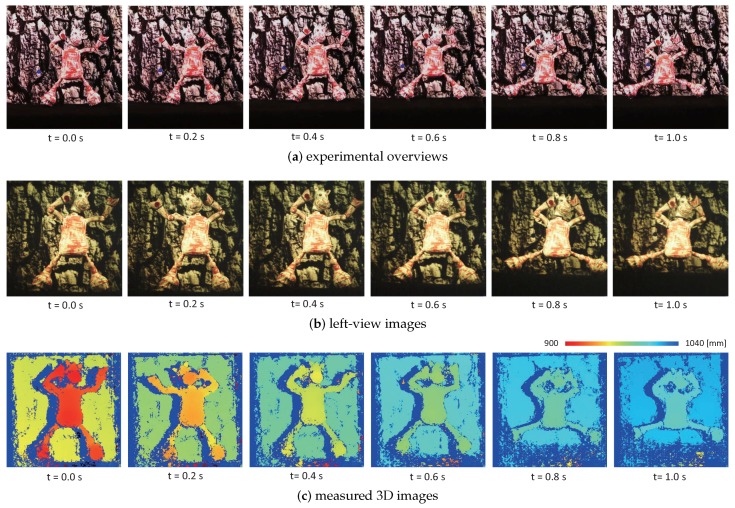
(**a**) Experimental overviews; (**b**) captured left-view images; and (**c**) measured 3D images of a dancing doll.

**Figure 18:**
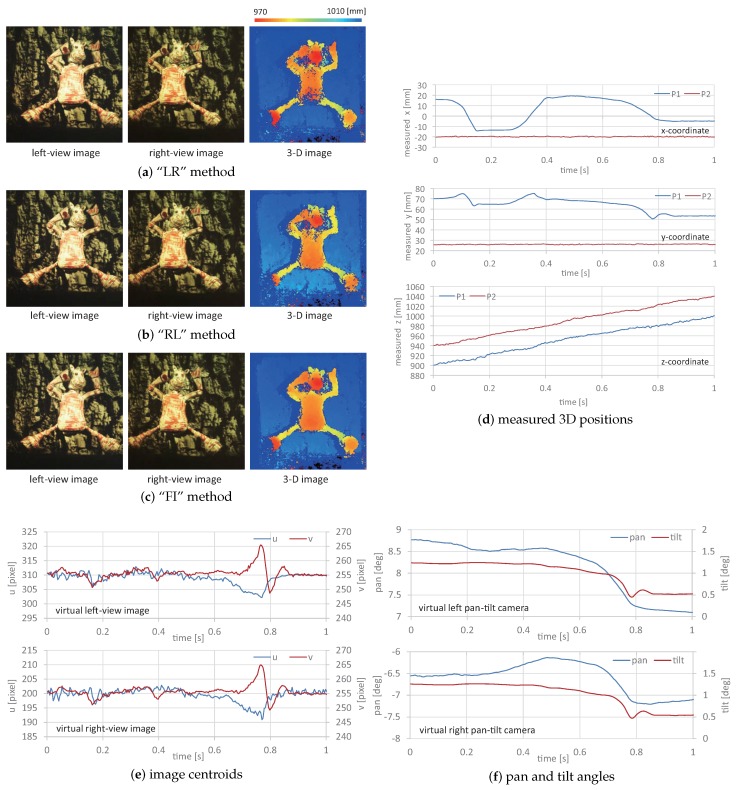
Measured 3D images and positions, image centroids, and pan and tilt angles of virtual left and right pan-tilt cameras when observing a dancing doll: (**a**) “LR” method; (**b**) “RL” method; (**c**) “FI” method; (**d**) measured 3D positions; (**e**) image centroids; (**f**) pan and tilt angles.

Figure 17 shows (a) a sequence of the experimental overviews, monitored using a standard video camera at a fixed position; (b) a sequence of the left-view images; and (c) a sequence of the estimated 3D images with virtual synchronization (“FI” measurement), respectively, which are taken at intervals of 0.2 s. Figure 18 shows (a)–(c) the left and right-view images and the 3D images in the “LR”, “RL”, and “FI” measurements at t= 0.712 s when the body of the doll quickly moved from up to down with the right-to-left motion of its front legs; (d) the 3D positions of the points $P_{1}$ and $P_{2}$, (e) the image centroids, and (f) the pan and tilt angles of the left and right pan-tilt cameras in the “FI” measurement for 1 s. The points $P_{1}$ and $P_{2}$ were located on the leg of the dancing doll and its background plane, respectively, as illustrated in Figure 16.

Figure 17 shows that the 3D shapes of the doll’s legs, which were cylinder-shaped with 5 mm diameter, were accurately measured when the legs moved quickly at different speeds from those around its other parts, whereas the body of the doll was so controlled to its desired value (310,255) in the left-view images that the *z*-coordinate values of the whole scene increased according to the linear slider’s motion from *z* = 900 to 1000 mm in the *z*-direction. In Figure 18d, the *x*- and *y*-coordinate values measured at the point $P_{2}$ were always around x=−20 mm and y= 26 mm, respectively, and its *z*-coordinate values varied from z= 940 to 1040 mm in a second when the linear slider moved at 100 mm/s in the *z*-direction; the difference between the *z*-coordinate values measured at the points $P_{1}$ and $P_{2}$ was always around 40 mm, corresponding to the 40-mm distance between the doll and the background plane. The *x*-coordinate values measured at the point $P_{1}$, which was located on the shaking leg of the doll, varied periodically in the range of x= −14 to 19 mm, and its *y*-coordinate values decreased from y= 70 to 54 mm according to the up-to-down motion of its body. In Figure 18e,f, the pan and tilt angles of the left and right pan-tilt cameras were so controlled that the image centroids in the left- and right-view images were held to around their desired values (310,255) and (200,255), respectively, when the dancing doll was moved in the *z*-direction by the linear slider. For t= 0.6–0.8 s when the body of the doll quickly moved from up and down, the image centroids in the left and right-view images slightly deviated from their desired values; these deviations corresponded to the tracking errors when the pan-tilt mirror system could not perfectly track the quick motion of the doll’s body in the *y*-direction. In Figure 18a–c, the 3D shapes in “LR”, “RL”, and “FI” measurements were similarly obtained, whereas the numbers of unmeasurable pixels in the 3D images in the “LR” and “RL” measurements were larger than that in the “FI” measurement. This is because the deviation errors were within 1 mm in the “LR” and “RL” measurements without virtual synchronization when the slider speed was 100 mm/s in the range of z= 900 to 1000 mm as illustrated in Figure 15e, whereas stereo correspondence was somewhat uncertain or inaccurate in the “LR” and “RL” measurements, due to the vertical displacement between the unsynchronized left and right-view images when capturing the doll moving in the *y*-direction.

These experimental results indicate that our catadioptric stereo tracking system can accurately measure the 3D shapes of time-varying-shape objects that have local parts at different speeds, while the target object is always tracked at the desired positions in the left and right-view images.

## 6. Conclusions

In this study, we implemented a catadioptric stereo tracking system for monocular stereo measurement by switching 500 different-view images in a second with an ultrafast mirror-drive pan-tilt camera. It can function as two virtual left and right pan-tilt cameras for stereo measurement that can capture a stereo pair of 8-bit color 512 × 512 images at 250 fps. Several 3D measurement results were evaluated using the high-frame-rate videos, which were being stored in stereo tracking with multithread gaze control; this evaluation verified the effectiveness of monocular stereo measurement using our catadioptric stereo tracking system with ultrafast viewpoint switching. Synchronization errors in monocular stereo measurement can be reduced by virtually synchronizing the right-view image with the frame-interpolated left-view image.

Currently, there are some problems to be solved: (1) insufficient light intensity due to the small-size mirror for ultrafast viewpoint switching; and (2) offline 3D image estimation due to the heavy computation required. To implement more sensitive and real-time stereo vision for real-world applications such as SLAM problems and large-scale mapping, we intend to improve both the optical system that maximizes the collection efficiency of light and the integrated monocular stereo algorithm that can be accelerated by parallel implementation of a local method with low computational complexity on GPUs and FPGAs. This is achieved by considering a trade-off between the complexity and accuracy in estimating stereo disparity maps on the basis of the monocular catadioptric stereo tracking system reported in this study.

## Figures and Tables

**Table 1:** Pros and cons of stereo vision techniques and active stereo systems.

	Stereo Vision Techniques		Active Stereo Systems	
classification	local methods [15,16,17,18,19,20,21]	global methods [9,10,11,12,13,14]	single pan-tilt mechanism [31,34]	multiple pan-tilt mechanisms [32,33]
calibration	direct calibration (such as Zhang’s method [46]) Pros: high calibration precision Cons: suitable for fixed stereo system		self-calibration [41,43,44 Pros: automatic parameter acquisition Cons: complex theory and parameter control LUT-based calibration [33,38] Pros: easy on-line parameter acquisition Cons: complex preprocessing for LUT feature-based calibration [39,40,42] Pros: parameters estimated by image features Cons: time-consuming and imprecise	
advantages	efficient for stereo matching and less time-consuming	accurate matching particularly for ambiguous regions	easy stereo calibration and gaze control	flexible views and extensive depth range
disadvantages	sensitive to locally ambiguous regions	very time-consuming	fixed baseline and limited depth range	real-time stereo calibration and complex gaze control

**Table 2:** Pros and cons of catadioptric stereo systems, fixed camera systems, and catadioptric stereo tracking system.

	Catadioptric Systems	Fixed Camera Systems	Catadioptric Stereo Tracking System
classification	planar mirror [53,54,55,56,57,58,59,70] bi-prism mirror [60,61] convex mirror [62,63,64,65,66,67,71,72,73,74,75]	lens aperture based [50,76,77], coded aperture based [51,52]	our proposed method
advantages	multi-view/wide field of view (convex)/no synchronization error/single camera	compact structure/rapid viewpoint-switching/easy calibration/single camera	active stereo/full image resolution/multi-view tracking/single camera
disadvantages	image distortion (convex)/half image resolution (planar or bi-prism)/inactive stereo	narrow baseline/limited field of view/synchronization errors/inactive stereo	insufficient incident light/synchronization errors/complex stereo calibration.
